# RandomizEd controlled trial for pre-operAtive dose-escaLation BOOST in locally advanced rectal cancer (RECTAL BOOST study): study protocol for a randomized controlled trial

**DOI:** 10.1186/s13063-015-0586-4

**Published:** 2015-02-22

**Authors:** JP Maarten Burbach, Helena M Verkooijen, Martijn Intven, Jean-Paul JE Kleijnen, Mirjam E Bosman, Bas W Raaymakers, Wilhelmina MU van Grevenstein, Miriam Koopman, Enrica Seravalli, Bram van Asselen, Onne Reerink

**Affiliations:** Department of Radiation Oncology, University Medical Center Utrecht, Heidelberglaan 100, 3584 CX Utrecht, The Netherlands; Trial Bureau Imaging Division, University Medical Center Utrecht, Heidelberglaan 100, 3584 CX Utrecht, The Netherlands; Department of Surgery, University Medical Center Utrecht, Heidelberglaan 100, 3584 CX Utrecht, The Netherlands; Department of Medical Oncology, University Medical Center Utrecht, Heidelberglaan 100, 3584 CX Utrecht, The Netherlands

## Abstract

**Background:**

Treatment for locally advanced rectal cancer (LARC) consists of chemoradiation therapy (CRT) and surgery. Approximately 15% of patients show a pathological complete response (pCR). Increased pCR-rates can be achieved through dose escalation, thereby increasing the number patients eligible for organ-preservation to improve quality of life (QoL). A randomized comparison of 65 versus 50Gy with external-beam radiation alone has not yet been performed. This trial investigates pCR rate, clinical response, toxicity, QoL and (disease-free) survival in LARC patients treated with 65Gy (boost + chemoradiation) compared with 50Gy standard chemoradiation (sCRT).

**Methods/design:**

This study follows the ‘cohort multiple randomized controlled trial’ (cmRCT) design: rectal cancer patients are included in a prospective cohort that registers clinical baseline, follow-up, survival and QoL data. At enrollment, patients are asked consent to offer them experimental interventions in the future. Eligible patients—histologically confirmed LARC (T3NxM0 <1 mm from mesorectal fascia, T4NxM0 or TxN2M0) located ≤10 cm from the anorectal transition who provided consent for experimental intervention offers—form a subcohort (*n* = 120). From this subcohort, a random sample is offered the boost prior to sCRT (*n* = 60), which they may accept or refuse. Informed consent is signed only after acceptance of the boost. Non-selected patients in the subcohort (*n* = 60) undergo sCRT alone and are not notified that they participate in the control arm until the trial is completed.

sCRT consists of 50Gy (25 × 2Gy) with concomitant capecitabine. The boost (without chemotherapy) is given prior to sCRT and consists of 15 Gy (5 × 3Gy) delivered to the gross tumor volume (GTV). The primary endpoint is pCR (TRG 1). Secondary endpoints include acute grade 3–4 toxicity, good pathologic response (TRG 1-2), clinical response, surgical complications, QoL and (disease-free) survival. Data is analyzed by intention to treat.

**Discussion:**

The boost is delivered prior to sCRT so that GTV adjustment for tumor shrinkage during sCRT is not necessary. Small margins also aim to limit irradiation of healthy tissue. The cmRCT design provides opportunity to overcome common shortcomings of classic RCTs, such as slow recruitment, disappointment-bias in control arm patients and poor generalizability.

**Trial registration:**

The Netherlands Trials Register NL46051.041.13. Registered 22 August 2013. ClinicalTrials.gov NCT01951521. Registered 18 September 2013.

## Background

Colorectal cancer is the second most common cancer in women and third in men worldwide [1]. Almost one-third of Dutch colorectal cancers are located in the rectum [2]. Rectal cancers are treated by surgery, preceded by chemoradiation in case of locally advanced rectal cancer (LARC). Chemoradiation consists of a total dose of about 50 Gy combined with capecitabine. With this treatment regimen, approximately 15% of patients show a pathological complete response (pCR) [3,4], classified by Mandard *et al*. as tumor regression grade 1 (TRG 1) [5]. In a recent meta-analysis, we showed that doses of ≥60 Gy were associated with an increased pCR rate to 20.4%, without compromising toxicity [6].

Patients with ‘good’ clinical response, either pCR (TRG 1) or near-pCR (TRG 2), might be eligible for organ-preserving approaches. The aim of these strategies is to deliver an optimal quality of life (QoL) without compromising the oncologic outcome in this favorable subgroup. Recently, several protocols have been developed, that use either local excision [7-9] or a “watch-and-wait” policy [10-12], in order to omit surgery (total mesorectal excision) as the primary treatment. In addition, patients who reach a pCR often show reduced local recurrence and improved (disease-free) survival probabilities compared with patients with a poor response (TRG 3–5) [3,13-15], possibly driven by a favorable tumor biology.

Thus, by escalating the preoperative radiation dose, the amount of patients with a ‘good’ clinical response or pCR who are eligible for organ-preserving treatment can potentially be increased. However, response rates after high-dose external radiation with 65 Gy have not yet been investigated in a randomized setting. Therefore, we set up an exploratory trial, the RandomizEd Controlled Trial for Pre-operAtive Dose-escaLation BOOST in Locally Advanced Rectal Cancer (RECTAL BOOST study: ClinicalTrials.gov NCT01951521) to compare tumor pCR rates (TRG 1), pathologic ‘good’ responses, toxicity levels, clinical tumor response and QoL between patients treated with 65 Gy chemoradiation (15 Gy boost plus 50 Gy chemoradiation) and those treated with 50 Gy standard chemoradiation therapy (sCRT).

## Methods/design

### Study design

This study is being conducted within the ProspectIve data CollectioN Initiative on Colorectal cancer (PICNIC) project [16]. The prospective observational PICNIC cohort includes patients with colorectal cancer of all stages. Information collected includes baseline demographic and clinical data, as well as prospective clinical follow-up and patient-reported outcome measures (PROMs). The study follows the cohort multiple randomized controlled trial (cmRCT) design [17] and provides a pragmatic infrastructure for multiple randomized controlled trials (RCTs).

### Patient recruitment

At enrollment in the PICNIC cohort, patients are asked to provide informed consent for prospective collection of clinical, survival and PROMs data. In addition, according to the cmRCT design, we ask patients’ consent to be randomly selected to receive offers on experimental interventions in the future and to use their data comparatively within the context of the PICNIC project.

From among the PICNIC cohort, we will identify all patients eligible for the boost intervention based on the following inclusion criteria: (1) histologically confirmed LARC, defined as T3 with threatened mesorectal fascia (<1 mm), T4 or N2M0 [18] (based on Dutch guidelines for chemoradiation in rectal cancer [19], in which N2 is defined as ≥4 positive nodes visible with diameter >9 mm or 5 to 9 mm combined with 2 of the following 3 characteristics: irregular border, heterogeneous or round-shaped); (2) tumor located ≤10 cm from the anorectal transition; and (3) previously obtained informed consent to be randomly offered experimental interventions within the context of the PICNIC project (Table 1). Patients are ineligible in case of inflammatory bowel disease, pregnancy, previous radiation to the pelvis, contraindication for capecitabine and inadequate comprehension of the Dutch language in speech and/or writing. Female patients in whom the tumor is located on the anterior wall close to the vagina are also ineligible because the maximum tolerated dose to the vagina does not allow for dose escalation when the tumor is in proximity to it.

**Table 1:** **Inclusion and exclusion criteria for the RECTAL BOOST study** ^**a**^

**Inclusion criteria**	**Exclusion criteria**
Participant in the PICNIC project	Metastatic disease
Informed consent obtained for being offered experimental interventions within the PICNIC project	Inflammatory bowel disease
Informed consent obtained for questionnaires on patient-reported outcomes within the PICNIC project	Prior radiation to the pelvis
Tumor distance of ≤10 cm from anorectal transition	Inadequate understanding of the Dutch language in speech and/or writing
Indication for chemoradiation based on Dutch guidelines	Recent pregnancy ≤1 yr ago
No contraindication for MRI	No indication for chemoradiation based Dutch guidelines
World Health Organization performance status 0–2	At least one contraindication for capecitabine administration

^a^RECTAL BOOST, RandomizEd Controlled Trial for Pre-operAtive Dose-escaLation BOOST in Locally Advanced Rectal Cancer; PICNIC, ProspectIve data CollectioN Initiative on Colorectal cancer; MRI, Magnetic resonance imaging.

### Random selection

**Figure 1:**
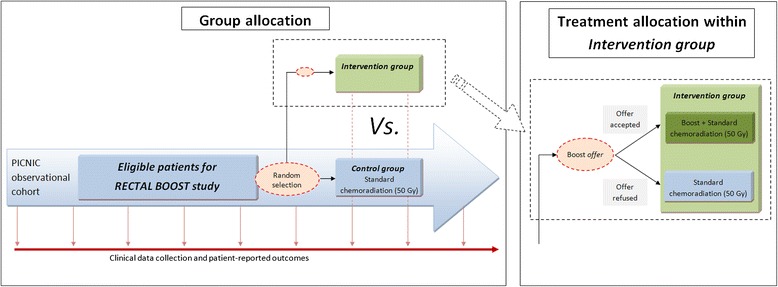
**Study design and flowchart.** PICNIC, ProspectIve data CollectioN Initiative on Colorectal cancer; RECTAL BOOST, RandomizEd Controlled Trial for Pre-operAtive Dose-escaLation BOOST in Locally Advanced Rectal Cancer.

Patients within the PICNIC cohort who meet the above inclusion criteria form a subcohort of eligible patients (Figure 1). From among this subcohort, a random sample is selected on a 1:1 basis with varying block sizes (*n* = 6 to 8) using a centrally available computer program. Randomly selected patients are offered the experimental intervention (boost prior to sCRT) by their treating physician. If they accept the offer, they will sign an additional informed consent to receive the boost. Patients who refuse the boost will receive care as usual (that is, sCRT). Patients in the subcohort who will not be randomly selected will not be informed about the boost intervention, nor will they be informed about their participation in the control arm of this study. When the trial is completed, aggregate disclosure about the trial results will be provided to the entire PICNIC cohort at the same time.

### Standard treatment

**Figure 2:**
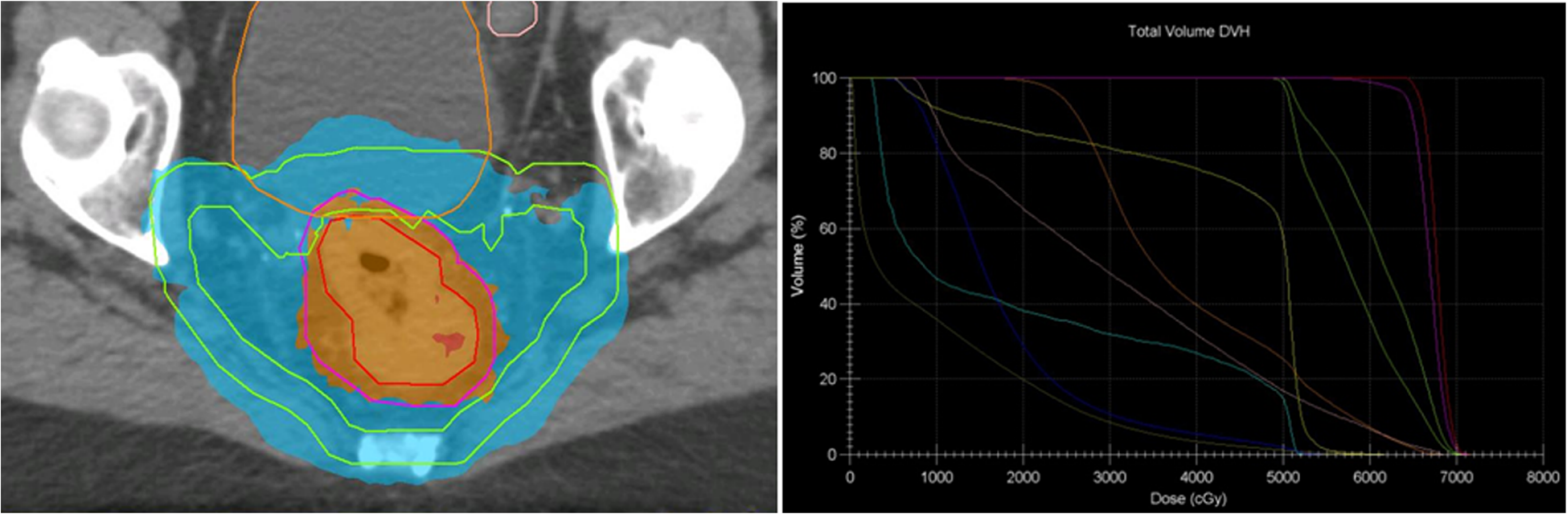
**Cumulative dose plan of boost plus standard chemoradiation fractions (left) with associated dose volume histogram (right).** Delineations of gross tumor volume (GTV) plus planned target volume (PTV_GTV_) (red), clinical target volume (CTV) plus PTV (PTV_CTV_) (green), bladder (brown) and sigmoid (pink). Areas that receive a total dose of >65 Gy are represented in orange), and those that receive >50 Gy are shown in turquoise.

sCRT consists of 50 Gy (25 × 2 Gy on weekdays) combined with capecitabine. The radiation dose is delivered by intensity-modulated radiation therapy (IMRT), which is standard care in our hospital, to the planned target volume (PTV), which comprises the gross tumor volume (GTV) and clinical target volume (CTV). Target volumes are delineated on computed tomography (CT) scans, matched to- and combined with T2- and diffusion-weighted magnetic resonance imaging (DWI) according to published guidelines [20]. The CTV follows the mesorectal fascia up to the rectosigmoid curvature and stretches maximally to 4 cm caudal from the tumor, sparing the sphincter in case of a low anterior resection (LAR), or includes the sphincter complex +1-cm margin in case of an abdominoperineal resection (APR). The lymph node regions (internal iliac and obturator) stretch from the caudal end of the v. iliaca communis downward to the crossing of the internal iliac vessels under the m. piriformis, laterally limited by the pelvic muscles. The obturator region stretches from the m. obturatorius to the m. levator, ventrally limited by the ureter or dorsal side of the neurovascular bundle without inclusion of the vesiculae, uterus and vagina. Lateral and dorsal border are marked by the pelvic muscles and ventral iliac region. In addition, the presacral region stretches from the upper level of the iliac vessels to mesorectum, ventrally limited 2 cm from the sacrum, including the a. rectalis superior and excluding the neuroforamina. The PTV_CTV50_ is a non-uniform margin around the CTV according to local protocol consisting of an expansion of 13 mm ventrally, 9 mm dorsally, 10 mm laterally and 10 mm craniocaudally (Figure 2). The prescribed dose to the PTV_CTV50_ is that 95% of the prescribed dose should cover ≥99% of the PTV. Radiation is delivered with an external beam linear accelerator. For position verification, the sCRT protocol consists of cone-beam CT prior to the first three fractions and weekly thereafter.

Capecitabine is administered orally twice daily on treatment days at a dose of 825 mg/m^2^, taken 2 hours before each radiation fraction and 12 hours later. Hematologic toxicity is tested every 2 weeks. Surgery is performed 10 to 12 weeks postradiation according to the Dutch guidelines [19]. The decision whether to perform total mesorectal excision surgery in form of LAR or APR, is made on the basis of the location and extensiveness of the tumor [21].

Two routine MRI scans are acquired using an MRI protocol developed in house, consisting of T2-weighted and DWI image sequences [22]. The first scan (taken maximally 2 weeks prior to radiation) is used for radiation planning, and the second (obtained 9 weeks postradiation) is used for preoperative response evaluation and surgical planning. Immediately prior to surgery, the surgeon will also perform a digital rectal examination (DRE) for clinical response evaluation.

### Boost intervention

The experimental boost intervention consists of 15 Gy (5 × 3 Gy on weekdays) to the GTV without concomitant chemotherapy. The boost is delivered in the week prior to the start of sCRT. A cumulative GTV dose of 65 Gy is delivered over the full treatment course of 30 fractions (6 weeks). This results in an equivalent dose in 2-Gy fractions of 66.3 Gy (α/β = 10 Gy) [23]. GTV delineation is based on T2-weighted and DWI scans; no CTV_GTV65_ margin is applied; and the PTV_GTV65_ is defined by a non-uniform margin of +11 mm in the anteroposterior direction, +7 mm in the lateral direction and +13 mm in the craniocaudal direction around the GTV, which represents tumor movement that was observed on in-house daily MRI scans and setup errors [24]. A volumetric modulated arc therapy stereotactic treatment plan is generated for boost patients, whereas IMRT is standard care used in the control arm, that accumulates the boost and standard chemoradiation doses (Figure 2). The maximally allowed cumulative dose within the GTV is 80 Gy (123% of 65 Gy), and the volume receiving 95% of the prescribed dose should be larger than 99% of the PTV_GTV65_. The maximum dose around, and dose prescription to, the PTV_CTV50_ is similar to the elective sCRT field (that is, 53.5 Gy). Daily online cone-beam CT scans are used for positioning before each boost fraction, and the sCRT position verification protocol follows thereafter.

Organs at risk (OARs) consist of the bowel bag (excluding sigmoid), bladder, vagina and anal sphincter. The dose constraints for the OARs remain unchanged for boost patients (namely, the dose to 1 cc of the OARs should be ≤53.5 Gy), and the volume receiving 45 Gy is aimed to be less than 195 cc for the bowel bag (excluding the sigmoid). OAR constraints are leading over PTV_GTV65_ coverage and may thus limit GTV dose if necessary. If dose prescriptions cannot be reached, a panel of radiation oncologists will discuss the feasibility and anticipated safety of the treatment plan and decide either to continue or to adapt the plan toward acceptable OAR constraints, taking into account anatomical limitations and planned surgery. One additional MRI scan is obtained in patients who receive the boost for response prediction purposes at the end of the second week (after ten fractions including the five boost fractions) and a rectoscopy for clinical response evaluation is performed in a subgroup of boost-arm patients that showed a ‘good’ response based on MRI.

### Primary endpoint

The primary endpoint of this study is pCR, which is classified according to the Mandard classification system as tumor regression grade 1 (TRG 1) [5] (that is, a sterile specimen with absence of residual cancer cells). Experienced gastrointestinal pathologists use a standardized protocol to evaluate the specimens [25], and central review of pathology is performed.

### Secondary endpoints

Secondary endpoints include non-complete pathologic responses (TRG 2–5), acute grade 3–4 toxicity, clinical response, surgical complications, QoL and disease-free and overall survival. The non-complete pathologic responses are categorized as good (TRG 1–2) or ‘not good’ (TRG 3–5) by the pathologist. A radiation oncologist will assess toxicity at weekly visits during the radiation treatment, as well as 4 weeks and 4 months after completion of sCRT. Toxicity is recorded according to the National Cancer Institute’s Common Terminology Criteria for Adverse Events, version 4.03 [26]. Long-term toxicity, (serious) adverse events (SAEs), hospitalization and other health-related problems are registered within the context of the PICNIC cohort during clinical follow-up and annual patient-administered questionnaires on health and oncological status. Clinical response evaluation is based on MRI (9 weeks after completion of chemoradiation; that is, the week before surgery) and DRE and rectoscopy, which are both performed right before surgery while the patient is under anesthesia. MRI and DRE are standardly performed in all patients, whereas rectoscopy is only performed in intervention arm patients who showed a good response based on MRI. For the MRI, a combination of T2-weighted and DWI sequences is used to measure residual tumor tissue in the initially delineated tumor region and its surrounding elective radiation field. Surgical complications are registered up to 30 days after the primary surgery in all patients and after closure of the diverting stoma in patients who previously underwent a LAR. For safety reasons, occurrence of anastomotic leakage is monitored closely in the LAR subgroup by daily review of postoperative patient charts for 30 days. QoL data are recorded by means of either online forms (Patient-Reported Outcomes Following Initial treatment and Long-term Evaluation of Survivorship (PROFILES): http://www.profilesregistry.nl/) or pencil-and-paper questionnaires. For this purpose, cancer-specific QoL questionnaires from the European Organization for Research and Treatment of Cancer (EORTC) are used, including the core (QLQ-C30 [27]) and colorectal cancer-specific (QLQ-CR29 [28,29]) questionnaires. The EORTC QLQ-C30 covers five functional scales, three symptom scales, a global QoL scale and six single items. The EORTC QLQ-CR29 assesses urinary, bowel and/or stoma, psychological and sexually related QoL issues, as well as side effects due to chemotherapy. These questionnaires are provided at the time of diagnosis (baseline) and 3, 6, 12, 18 and 24 months thereafter. Survival and disease-free survival are monitored within the PICNIC project through clinical follow-up records and via a link with the Dutch Cancer Registry. Disease-free survival is defined as the time in absence of a rectal cancer local recurrence or metastasis.

### Safety

According to Dutch law, the investigator reports SAEs within 15 days following notification through a government-based internet portal (Centrale Commissie Mensgebonden Onderzoek (Central Committee on Research Involving Human Subjects): https://www.toetsingonline.nl/) to the accredited institutional review board (IRB) that approved the protocol. SAEs that result in death or are life-threatening will be reported within 7 days.

Because this is an exploratory dose escalation study, an independent data and safety monitoring board (DSMB) will make recommendations on continuation of the study based on safety results, focusing on toxicity and anastomotic leakage. The DSMB consists of an expert surgeon, a radiation oncologist and a statistician and is provided, annually or after every SAE, with the raw data on the primary and secondary outcomes (including toxicity, surgical complications and survival). After the first 10 patients with LAR have undergone boost treatment, inclusion of patients scheduled for LAR will be stopped for 8 months to let the DSMB compare anastomotic leakages between boost and control arms. The 8-month period will consist of the time between sCRT to primary surgery (10–12 weeks) followed by the time between primary surgery and bowel reconstruction, including a postoperative monitoring period (16–20 weeks). Only inclusion of patients undergoing LAR will be halted during this stop, because patients undergoing APR have no risk of anastomotic leakage, owing to their permanent stoma. The DSMB analyzes the data independently of the investigators and reports their advice on continuation of the study to the sponsor, which will decide on continuation or stopping of the study.

### Sample size considerations

On the basis of our center’s experience, we assume that 13% of patients will reach pCR if undergoing sCRT. On the basis of a prediction model published by Appelt *et al.* [30], we expect the pCR rate to be 30% after 65 Gy treatment. Because we consider this study to constitute preliminary work for subsequent studies aimed at evaluating even higher dose increases, we deem it important to find an effect if there really is one, but less important to unjustly find an effect. Therefore, we will use a one-sided α of 15% because it is unlikely that the pCR rate after boost treatment plus sCRT will be lower than after sCRT alone, in combination with a power of 80% because we do not want to increase uncertainty when a negative result is achieved. We further expect that approximately 80% of the patients who receive a boost offer will accept it. Patients who are offered the boost treatment but refuse to undergo the boost will remain in the intervention arm for analysis but receive sCRT (Figure 1). We expect no cross-over from the control arm to the intervention arm, because only patients who are randomly selected to receive a boost offer are informed about and offered it, whereas all non-selected patients undergo standard treatment (that is, sCRT) without receiving information about the boost trial. Taking into account the estimated response rates, together with a 20% refusal rate in the intervention arm, we require 60 patients per arm to demonstrate a statistically significant difference. We expect to complete recruitment within 3 years.

### Data analysis

Data will be analyzed according to the intention-to-treat principle. Data of eligible patients who were randomly offered the boost (intervention arm) will be compared with eligible patients who were not randomly selected (control arm). In case of dropout (that is, no surgery following chemoradiation), a worst-case analysis will be performed in which all non-resected intervention-arm patients are classified as non-complete responders and all non-resected control patients are classified as complete responders. However, because omission of surgery is not common practice in our institution, we expect these numbers to be small. In case of substantial boost treatment refusal in the intervention arm, complier average causal effect analysis will be performed to deal with differences in compliance with treatment between both arms [31]. Outcomes include tumor pCR rate (TRG 1), good pathological response (TRG 1–2), clinical response, grade 3–4 toxicity, QoL, recurrence rates and disease-free and overall survival.

The primary outcome (proportion of patients with pCR) will be presented in proportions and compared by means of the χ^2^ test. Toxicity will be presented as the overall and/or time point-specific incidence of grade 3–4 toxicity, and differences will be tested with the χ^2^ test. QoL will be compared at multiple points in time. A relevant change in the patient’s perspective is indicated by a 10% difference or more than 0.5 of the standard deviation [32]. QoL data will be analyzed by mixed-effects models. Differences in disease-free and overall survival rates will be analyzed by Kaplan-Meier analysis and log-rank test. Differences with a *P*-value <0.05 are considered statistically significant, except for the primary endpoint, where *P* < 0.15 has been prespecified.

### Ethical approval

IRB approval was obtained separately for both the PICNIC project (including the cmRCT infrastructure) and the RECTAL BOOST study from the ethical body of the UMC Utrecht (under reference numbers 12/510 and 13/522, respectively). The PICNIC project is published under NCT02070146 [16] and the RECTAL BOOST study under NCT01951521 [33] on ClinicalTrials.gov.

## Discussion

The RECTAL BOOST study aims to quantify the effect of an external beam radiation (EBR) boost of 15 Gy prior to standard chemoradiation (50 Gy) on pCR rates in patients with locally advanced rectal cancer. Toxicity, clinical (complete) response, (surgical) complications, (disease-free) survival, QoL and feasibility of boost delivery are secondary endpoints. In this study, we will assess in a randomized fashion whether a preoperative 65 Gy EBR-only regimen (15 Gy boost followed by 50 Gy chemoradiation) can safely increase the proportion of patients with pCR (TRG 1) in comparison with the proportion observed after 50 Gy chemoradiation alone. Although contact X-ray [34], brachytherapy, EBR and EBR-brachytherapy combined studies [6] have shown response, sphincter-saving and organ preservation benefits at doses up to 60 Gy, such benefits have not been shown for the 65 Gy dose level when reached by an EBR-only approach.

High rates of good responders are important, as this will increase the number of patients eligible for organ-preserving treatment strategies. These strategies aim to improve the QoL of patients with a good response while their good oncologic outcome, and possible survival benefits, are maintained. Nevertheless, the value of tumor regression has not yet been confirmed as a surrogate endpoint for oncologic outcome in this setting. In the present study, however, all patients, including those with a good clinical response to preoperative treatment, will still undergo surgery because pathological response is the primary endpoint, and because resection remains the standard care according to Dutch guidelines [19]. Therefore, future randomized studies in patients with good clinical responses after (boost) chemoradiation are needed to further assess the effect of radical surgery versus organ preservation on QoL, toxicity and (disease-free) survival. In this study, we aim to quantify response rate by its current gold standard (that is, pathology), which in turn can be correlated to the clinical response data that are also obtained. In so doing, this study will provide important insight into the results of dose escalation that would otherwise be lacking in a trial that would combine dose escalation and omission of surgery within the same trial.

In this study, we choose to deliver the boost prior to chemoradiation to obtain maximal tumor visibility in order to avoid dose administration to healthy tissue. The rationale underlying this is that GTV deformation is likely to occur during sCRT as a result of tumor shrinkage, and because the induced inflammatory reaction results in edema which hinders adequate GTV definition. Both factors make imaging and delineation of the tumor more difficult and less reliable thereby likely to result in overestimation of the remaining GTV. When larger areas would then incorrectly be irradiated, surrounding healthy tissue might receive a higher dose than planned. On the basis of our own data on patients receiving 5 × 5 Gy radiation within one week, we know that tumor shrinkage does not yet occur during the first week of radiation, which implies that GTV delineations made prior to irradiation remain adequate throughout the first week of (chemo)radiation. This allows a single GTV delineation to be used for boost dose planning in the week prior to sCRT. Furthermore, we choose to use IMRT because this is the standard of care in our hospital, and this study is set up as a single-facility trial. Direct applicability of these results should thus be considered when extrapolated to other centers in which different radiation techniques are used.

We have chosen to apply the cmRCT design, which aims to overcome common shortcomings of classic RCTs, such as slow recruitment, disappointment bias in patients randomized to the control arm, and poor generalizability. Not uncommonly, oncological patients possess strong preferences for experimental treatments that are (often) falsely regarded to be superior. This prevents such patients from taking part in randomized studies, thereby diminishing recruitment rates for RCTs. Furthermore, patients who remain willing to participate in RCTs often represent a younger, healthier, higher-educated subgroup, and once participating, these patients are unfortunately often allowed to participate in only one trial at a time. All this makes recruitment for RCTs difficult and prone to selection bias. Because the cmRCT design uses a cohort as a recruitment pool, it represents the routine population more adequately because cohort inclusion is generally less selective. Because this baseline may also evolve over time, the cmRCT furthermore provides the advantage that the effectiveness of experimental interventions is compared with the most up-to-date available standard care with which it should compete, instead of competing with outdated treatments which is often the case when classic RCTs are published. A new aspect brought by the cmRCT design is that it provides the opportunity to evaluate acceptance rates of offered treatments. This offers new insights into patient preferences and reasons for refusal of experimental interventions that become increasingly important. It forces clinicians and researchers to rethink their treatment approach at a much earlier stage when, for instance, a large proportion of patients declines the offered treatment. Overall, efficient, less selective recruitment, collection of long-term outcomes and early preference monitoring could reduce research costs significantly when conducting RCTs within the cmRCT design.

The RECTAL BOOST study is a pragmatic RCT performed within the infrastructure of the cmRCT design, that aims to quantify the effect on pCR rate of preoperative dose escalation to 65 Gy in comparison with standard 50 Gy chemoradiation in patients with locally advanced rectal cancer. If the proportion of good responders can be increased by dose escalation, this strategy could provide an option to increase the number of patients that may benefit from organ-preserving strategies in the future.

## Trial status

Ethical approval for this trial was obtained in June 2014. Recruitment started in September 2014 and is currently ongoing.

## Abbreviations

APR: Abdominoperineal resection
cmRCT: Cohort multiple randomized controlled trial
CRT: Chemoradiation treatment
CT: Computed tomography
CTV: Clinical target volume
DSMB: Data and safety monitoring board
DWI: Diffusion-weighted imaging
EBR: External beam radiation
EORTC: European Organization for Research and Treatment of Cancer
GTV: Gross tumor volume
IMRT: Intensity-modulated radiotherapy
LAR: Low anterior resection
LARC: Locally advanced rectal cancer
MRI: Magnetic resonance imaging
OAR: Organ at risk
pCR: Pathologic complete response, defined as Mandard grade 1 (TRG 1)
PICNIC: ProspectIve data CollectioN Initiative on Colorectal cancer
PROMs: Patient-reported outcome measures
PTV: Planned target volume
RCT: Randomized controlled trial
SAE: Serious adverse event
sCRT: Standard chemoradiation treatment
TRG: Tumor regression grade, as defined by Mandard classification
